# Survey instruments used in clinical and epidemiological research on waterpipe tobacco smoking: a systematic review

**DOI:** 10.1186/1471-2458-10-415

**Published:** 2010-07-13

**Authors:** Elie A Akl, Sohaib Aleem, Sameer K Gunukula, Roland Honeine, Philippe Abou Jaoude, Jihad Irani

**Affiliations:** 1 Department of Medicine, State University of New York at Buffalo, Buffalo, NY, USA; 2 Department of Family Medicine, State University of New York at Buffalo, Buffalo, NY, USA; 3 Department of Clinical Epidemiology and Biostatistics, McMaster University, Hamilton Canada; 4 Department of Social and Preventive Medicine, State University of New York at Buffalo, Buffalo, NY, USA; 5 Faculty of Health Sciences, University of Balamand, Beirut, Lebanon

## Abstract

**Background:**

The primary objective was to systematically review the medical literature for instruments validated for use in epidemiological and clinical research on waterpipe smoking.

**Methods:**

We searched the following databases: MEDLINE, EMBASE, and ISI the Web of Science. We selected studies using a two-stage duplicate and independent screening process. We included papers reporting on the development and/or validation of survey instruments to measure waterpipe tobacco consumption or related concepts. Two reviewers used a standardized and pilot tested data abstraction form to collect data from each eligible study using a duplicate and independent screening process. We also determined the percentage of observational studies assessing the health effects of waterpipe tobacco smoking and the percentage of studies of prevalence of waterpipe tobacco smoking that have used validated survey instruments.

**Results:**

We identified a total of five survey instruments. One instrument was designed to measure knowledge, attitudes, and waterpipe use among pregnant women and was shown to have internal consistency and content validity. Three instruments were designed to measure waterpipe tobacco consumption, two of which were reported to have face validity. The fifth instrument was designed to measure waterpipe dependence and was rigorously developed and validated. One of the studies of prevalence and none of the studies of health effects of waterpipe smoking used validated instruments.

**Conclusions:**

A number of instruments for measuring the use of and dependence on waterpipe smoking exist. Future research should study content validity and cross cultural adaptation of these instruments.

## Background

Waterpipe smoking is a traditional form of tobacco consumption in the region of the Middle East[1]. This form of smoking employs a device (the waterpipe) that heats tobacco using charcoal and then filters the smoke in a bowl of water before its inhalation through a rubber pipe[2]. There are regional variations in the type of tobacco smoked, and in the shape, the size, and the appearance of the waterpipe device[3].

Waterpipe smoking differs from other forms of tobacco consumption in a number of aspects. Its use can be intermittent (e.g. once per week) and the length of each session can vary from few minutes to few hours[4]. In addition, family members and friends typically use waterpipe during social gatherings[1]. Also, hookah cafes, an emerging trend in Western societies, represent a unique setting for waterpipe used. Finally, it is not unusual that different smokers share the same device, a practice that has been hypothesized to cause the transmission of communicable diseases [1].

There is increasing evidence supporting the deleterious health effects of waterpipe smoking [5]. A recent systematic review found that waterpipe tobacco smoking is possibly associated with a number of deleterious health outcomes such as lung cancer, esophageal cancer, respiratory illness, low birth weight and periodontal disease [5]. Similar associations may exist with bladder cancer, oral dysplasia, and coronary heart disease[5].

In spite of the possible harms of waterpipe smoking, its use has been increasing at alarming rates across countries. A recent literature review found that waterpipe tobacco smoking is increasing in prevalence worldwide with 10-20% prevalence in Arab American young adult populations in the United States [6].

A growing number of studies are investigating the prevalence of waterpipe tobacco smoking [6] as well as exploring its association with a number of health outcomes[5]. The validity of the results of these studies depends on the use of a validated instrument to measure waterpipe smoking. Indeed the World Health Organization (WHO) Tobacco Product Regulation (TobReg) issued back in 2005 a report on waterpipe tobacco smoking calling for research on methods for evaluating smoker exposure[7].

Our primary objective was to systematically review the medical literature for instruments validated for use in epidemiological and clinical research on waterpipe smoking. Our secondary objective was to determine the percentage of epidemiological and clinical studies that have used validated survey instruments.

## Methods

### Eligibility criteria

For the primary objective, we included papers reporting on the development and/or validation of survey instruments to measure waterpipe tobacco consumption or related concepts (e.g. waterpipe dependence) in epidemiological and clinical research;

For the secondary objective, we included reports of observational studies assessing the association between waterpipe tobacco smoking and relevant health outcomes or estimating the prevalence of waterpipe tobacco smoking.

We included paper reporting original data relating to our specific objectives even if waterpipe use was not the main subject (e.g. main subject was tobacco use).

### Search Strategy

We conducted a comprehensive search for studies relating to waterpipe use in June 2008 using no language restrictions. We searched the following electronic databases from their dates of inception: MEDLINE, EMBASE, and ISI the Web of Science. Additional file 1 provides the electronic search strategies that were based on a preliminary review of relevant articles, an Internet search for synonyms of waterpipe and a related published search strategy[8]. Two medical librarians commented on the search strategy. Additional search strategies included screening the lists of citations of included and relevant papers, and using the 'Related Articles' feature in PubMed.

### Selection process

In a first step, two reviewers screened in a duplicate and independent manner the title and abstract of identified citations for potential eligibility. We retrieved the full texts of citations that at least one reviewer judged as potentially eligible. In a second step, two reviewers used a standardized and pilot tested screening form to screen in a duplicate and independent manner the retrieved full texts. The two reviewers resolved their disagreements by discussion or by consulting a third reviewer.

### Data abstraction

Two reviewers abstracted data from each eligible study in a duplicate and independent manner using a standardized and pilot tested data abstraction form. Disagreements were resolved by discussion or by a third reviewer. We finally attempted to contact the corresponding authors of eligible studies asking them to verify our abstracted data and provide any additional relevant information.

For studies reporting on survey instruments to measure waterpipe tobacco consumption or related concepts, we extracted data relating to:

1. The name of the instrument and the concept being measured

2. Participants included in the development and validation of the instrument

3. The development methods

4. The validation methods

5. Additional relevant information

For studies of association of waterpipe use with clinical outcomes and of prevalence of waterpipe use, we assessed whether the study measured waterpipe use using:

1. An instrument with details not reported;

2. A self developed instrument, but no validation reported;

3. A self developed instrument, and validation reported;

4. A self developed instrument based on previously developed validated tool, but no validation of new instrument reported;

5. A previously developed instrument, but no validation reported;

6. A previously developed instrument, and validation reported.

## Results

### Survey instruments

We identified a total of five survey instruments: one to measure knowledge, attitudes, and waterpipe use among pregnant women,[9] three to measure waterpipe use,[4,10,11] and one to measure waterpipe dependence [12]. Additional file 2 provides detailed information about the instrument, the development process, the validation process, and the participants involved in these 2 processes. A brief description of each instrument follows.

#### Instrument by Chaaya et al

The questionnaire was developed for the assessment of knowledge, attitudes, and practices of waterpipe and cigarette use among pregnant women[9]. The authors generated the items from literature review and discussion with field workers. The instrument was assessed for internal consistency and content validity.

#### Instrument by Hanna et al

The questionnaire was designed for assessing the use of different forms of tobacco, including cigarette, cigar, bidi, pipe, smokeless tobacco, and waterpipe[10]. The investigators generated the items from previously developed and validated instruments from the UK and refined the instrument for linguistic, content, and social acceptability.

#### Instrument by Maziak et al

This instrument was designed for the assessment of waterpipe use[4]. It consists of 10 items about the status of waterpipe use (ever, current, former), the pattern of use and quitting. The authors generated the items through literature review and discussions among tobacco researchers. The authors did not report any pilot study or validation work.

#### Global adult tobacco survey (GATS)

The Global adult tobacco survey (GATS) is a product of the Global Tobacco Surveillance System (GTSS), a collaborative effort between The World Health Organization (WHO), the Centers for Disease Control and Prevention (CDC), and the Canadian Public Health Association (CPHA) funded by the Bloomberg global initiative[11]. GATS is a household survey to track prevalence, exposure to risk, second hand smoke, cessation, risk perception, knowledge and attitude, exposure to media and price as well as taxation issues with cigarette and other tobacco use. A waterpipe module consists of 6 core questions and 4 optional questions. The module was built with expert input then pretested in 4 countries. After translation and back translation, the instrument was fielded in the same 4 countries. Reproducibility and validity data are pending.

#### Lebanon Waterpipe Dependence Scale (LWDS-11)

The investigators designed the Lebanon Waterpipe Dependence Scale (LWDS-11) as an instrument for measuring waterpipe smoking dependence for both research and clinical purposes[12]. The instrument is composed of eleven scale items in four subscales measuring nicotine dependence (4 items), negative reinforcement (2 items), psychological craving (3 items), and positive reinforcement (2 items) (Table). Each item is scored on a 4-point Likert scale and a total score of 10 is used to indicate dependence. The development process consisted of item generation and item reduction (Table). In the validation process the investigators found adequate internal consistency, test-retest reproducibility, adequate convergent construct validity, and discriminant validity. The threshold score of 10 discriminated satisfactorily between mild, moderate, and heavy waterpipe smokers.

### Review of studies of association of waterpipe use with health outcomes

We identified 23 eligible studies assessing the association between waterpipe tobacco smoking and health outcomes (Additional file 3)[5]. Of these, 8 (35%) did not report any details about the instrument used and 15 (65%) reported using a self developed instrument with no validation reported. None of the studies reported using a previously developed validated instrument.

### Review of studies of prevalence of waterpipe use

We identified 38 eligible studies estimating the prevalence of waterpipe use (Additional file 4; unpublished data). Of these, 15 (40%) did not report any details about the instrument used; 11 (29%) reported using a self developed instrument with no validation reported; 10 (26%) reported using a self developed instrument based on previously validated instruments, with no validation of the new instrument reported; 1 (3%) reported using a previously developed instrument with no validation reported, and 1(3%) reported using a previously developed instrument with no validation reported for waterpipe smoking.

## Discussion

We identified a total of five survey instruments: one to measure knowledge, attitudes, and waterpipe use among pregnant women; three designed to measure waterpipe tobacco consumption; and one to measure waterpipe dependence. Rigorous development and validation processes were described only for the latter one. One prevalence study and no study on the health effects of waterpipe tobacco smoking have used validated instruments.

This study has a number of strengths. First, this is the first systematic review of instruments validated for use in epidemiological and clinical research on waterpipe smoking. Second, we used a rigorous methodology including a very sensitive and comprehensive search strategy, a duplicate and independent selection process, and a duplicate and independent data abstraction process. Unfortunately, we did not identify valid instruments to measure waterpipe smoking.

The different instruments have different strengths. The instrument by Chaaya et al. has been specifically developed for pregnant women and evaluates knowledge and attitude in addition to the use of waterpipe. The one by Hanna et al. enjoys a cross-cultural comparability through its translation into 4 languages. The GATS instrument was rigorously developed and is the most comprehensive in terms of measuring the different characteristics of waterpipe use (Table). However, reliability and validity data are still pending. LWDS-11 benefited from a rigorous development and validation processes and is the only instrument that measures dependence. It also measures the number of waterpipe smoked per week, felt pleasure and social component of the practice.

While the growing number of studies being conducted in the area of waterpipe tobacco smoking is very encouraging, their use of non validated tools is concerning. The bias potentially introduced by this use decreases our confidence in the estimates of associations of waterpipe smoking with health and in the estimates of its prevalence of use. It is important to note that all identified instruments have been developed relatively recently (published within the last 5 years); as these instruments are now available we should expect that more future studies will use validated tools.

Two important aspects of validity for instruments measuring variables such as waterpipe tobacco consumption are content validity and cross cultural adaptation. Content validity is important to ensure the coverage of all important aspects (e.g. the type of tobacco used, the concomitant use of other substances, the number of "rocks smoked"), patterns (e.g. the frequency of use, the number of years of use, and the number and duration of smoking sessions (nafas)), and status (e.g., ever, current, former) of waterpipe use. Cross cultural adaptation - through the development of different language versions and their testing in different settings [13] - is important given the higher prevalence of waterpipe use among specific ethic groups[6]. Such adaptation would need to be carefully conducted (e.g. with local participation) given that a simple 'professional' translation often fails to take into account common parlance.

Studies of prevalence of waterpipe smoking require instruments measuring the patterns of consumption. Hopefully these instruments will also help in standardizing the methods of reporting the prevalence (waterpipe only smokers versus all waterpipe smokers) and the status of waterpipe smoking (e.g. ever, current, former). The use of these standardized instruments will allow the assessment of both spatial and secular trends and the estimation of the burden of waterpipe smoking. Studies of the health effects of waterpipe tobacco smoking will need to use comprehensive instruments in order to capture the different patterns and aspects of waterpipe smoking that might be associated with the health outcome.

## Conclusion

Future work on survey instruments on waterpipe tobacco smoking needs to take into account content validity and cross cultural adaptation[13]. Details of instrument administration such as the time of completion, and the need for an interviewer would also be important for researchers in the field. Also, in order for such instruments to be incorporated into clinical practice, they should be validated in that setting and assessed for feasibility. Finally, researchers in the field should establish collaborative efforts to avoid duplication of work and develop a commonly accepted and used instrument.

## Competing interests

The authors declare that they have no competing interests.

## Authors' contributions

EAA contributed to drafting the protocol, designing the search strategy, developing the forms, screening, data abstraction, data analysis, and drafting of the manuscript. SA contributed to data abstraction, data analysis, and drafting of the manuscript. SG: contributed to data abstraction, data analysis and drafting the manuscript. RH contributed to screening and data abstraction. PAJ contributed to screening. JI contributed to drafting the protocol and designing the search strategy. All authors read and approved the final manuscript.

## Pre-publication history

The pre-publication history for this paper can be accessed here:

http://www.biomedcentral.com/1471-2458/10/415/prepub

## Supplementary Material

Additional file 1 **Electronic search strategies**. Provides the detailed search strategies used in the systematic review Click here for file

Additional file 2 **Identified survey instruments**. Describes the instruments, their development and their validation processes Click here for file

Additional file 3 **Validity of instruments in studies of health effects**. Describes the validity of instruments to measure waterpipe tobacco smoking used in studies assessing its effects on health outcomes Click here for file

Additional file 4 **Validity of instruments in prevalence studies**. Describes the validity of instruments to measure waterpipe tobacco smoking used in studies assessing its prevalence of use Click here for file
