# Supplementary material for: Survey instruments used in clinical and epidemiological research on waterpipe tobacco smoking: a systematic review
Source: BMC Public Health. 2010 Jul 13;10:415. doi: 10.1186/1471-2458-10-415 (PMC2912817; doi:10.1186/1471-2458-10-415)
Supplement: Additional file 2 — Identified survey instruments. Describes the instruments, their development and their validation processes [file 1471-2458-10-415-S2.DOC]

**Additional file 2**

| **Study ID** | **Instrument** | **Participants** | **Development** | **Validation** | **Additional information** |
| --- | --- | --- | --- | --- | --- |
| Chaaya 2004 [9] | - Questionnaire on practices of waterpipe and cigarette use among pregnant women (also measures knowledge and attitudes) - Language: Arabic - Availability: not published | - Not reported | - Item generation:   - literature review   - discussions with field workers for relevance to the population surveyed   - revised by the Ministry of Public Health - Pilot tested on 30 women - 120 questions including questions on actual cigarette and arguileh practices: current and previous use, smoking patterns (regular, occasional), age at initiation, place of smoking, smoking frequency, quitting attempts, and smoking status of husband and other household members. | - Internal consistency: Cronbach’s alpha:   - knowledge scale (α=0.91)   - attitude scale (α=0.85) - Content validity: inspection of previous similar questionnaires | Smokers are subdivided into:   - persistent smokers (women who continued smoking all through their pregnancy) - spontaneous quitters (women who successfully quit smoking due to their pregnancy) - failed quitters (women who stopped smoking for a while, but then relapsed) |
| Maziak 2005 [4] | - Questionnaire for the assessment of waterpipe use. - Language: English. - Availability: Appendix A of the paper [4] | - N/A | - Item generation:   - literature review   - discussions among teams working in tobacco research in East Mediterranean region - 10 items: ever smoking (1), current smoking (1), former smoking (1), pattern of use (5), quitting (2) | - Face validity | - No pilot study done |
| Hanna 2006 [10] | - Questionnaires on use of different forms of tobacco - Languages: Punjabi, Urdu, Sylheti Cantonese - Availability: <http://www.ashscotland.org.uk/ash/ash_display.jsp?pContentID=4385&p_applic=CCC&p_service=Content.show&> | - Four bilingual coworkers: a Pakistani, a Bangladeshi, a Chinese and an Indian Sikh. - Panel of 10 lay people, preferably monolingual for each of the languages of interest | - Item generation:   - Questions derived from 6 UK questionnaires, mainly the Health Survey for England 1999   - Questions from selected questionnaires already translated to the languages of interest.   - Translation of remaining questions by bilingual coworkers - Refinement for linguistic, content, and social acceptability with monolingual lay people:   - One to one consultation   - Panel discussions | - Face validity: field testing for acceptability and understanding with 20 subjects per language recruited by coworker (except Sylheti) - Cross-cultural comparability: literal back translation into English by coworkers; each question checked for equivalence and comparability to every other language and to English; where necessary, changes were made for comparability | - Key areas covered (vary by culture): cigarette, cigar, bidi, pipe, waterpipe, smokeless tobacco - Extremely difficult to recruit a Sylheti –English speaking coworker. Thus some phases of the research had to be omitted |
| Global adult tobacco survey (GATS) [11] | - Questionnaire for the assessment of waterpipe use. - Language: English, Arabic, Turkish, Ukrainian, and Vietnamese - Availability: <http://www.cdc.gov/tobacco/global/gats/> [11] | - Samples from Egypt, Turkey, Ukraine, and Vietnam | - 6 core questions: frequency of use, age at first use, number of years of use, duration of smoking session, sharing of waterpipe device - 4 optional questions: number of “rocks” smoked, location of use, use of flavored tobacco, use of other substances | - Consultation with 3 experts - Each country-specific questionnaire was translated into the local language(s), back translated into English, and then reviewed for appropriateness. - Pretested an fielded in the 4 aforementioned countries; reliability and validity data pending | - Detailed instructions on conducting the survey are available [11]. |
| Salameh 2008 [12] | - Lebanon Waterpipe Dependence Scale (LWDS-11) - Concept measured: waterpipe dependence - Language: Arabic - Availability: Table 2 of the paper [12] | - Sample 1   - convenience sample of 103 regular waterpipe smokers   - Face to face interview   - Semiquantitative measurement of nicotine metabolites - Sample 2:   - convenience sample of 15 regular waterpipe smokers   - Face to face interview   - Semiquantitative measurement of nicotine metabolites - Sample 3:   - random sample of 188 regular waterpipe smokers   - Telephone interview using random digital dialing | - Item generation: 21 items; 15 adapted from FTND & DSM-IV; 6 added by authors - Pretesting of preliminary version in 8 waterpipe smokers - Item reduction:   - face to face interview (sample 1)   - principal component analysis leading to 11 items questionnaire (sample 1) - Final version: 11 items in 4 subscales:   - Nicotine dependence (4)   - Negative reinforcement (2)   - Psychological craving (3)   - Positive reinforcement (2) | - Reproducibility : test retest 2 weeks apart (sample 1) (r=0.92) - Internal consistency: Cronbach’s alpha (α=0.83) - Construct validity: cross validation by principal components analysis (sample 3) - Discriminant validity: intersubscale correlation and component correlation matrix (sample 1,2 and 3) ((r<0.38) - Convergent construct validity: correlation between LWDS-11 scale and subscales with salivary cotinine, exhaled-air CO and the number of waterpipes per week (samples 1 and 2) ((0.71<r<0.90) - Group differentiation: between heavy, moderate, and mild smoker by LWDS-11 scoring, (samples 1 and 3) (p<0.0001) | - Scale: 4-point (0–3) Likert-type - Scoring: sum of subscales scores - Threshold for dependence: 10 - Semiquantitative measurement of nicotine metabolites:   - Exhaled CO measurement prior to the beginning of the smoking (samples 1, 2) - Nicotine metabolite measurements in saliva 1 hr after beginning of smoking (semiquantiative method for sample 1; HPLC quantitative method for sample 2) |

FTND = Fagerstrom Test for Nicotine Dependence

DSM-IV = Diagnostic and Statistical Manual of Mental Disorders of dependence

CO = Carbon monoxide

HPLC = high performance liquid chromatography
