# Supplementary material for: Survey instruments used in clinical and epidemiological research on waterpipe tobacco smoking: a systematic review
Source: BMC Public Health. 2010 Jul 13;10:415. doi: 10.1186/1471-2458-10-415 (PMC2912817; doi:10.1186/1471-2458-10-415)
Supplement: Additional file 3 — Validity of instruments in studies of health effects. Describes the validity of instruments to measure waterpipe tobacco smoking used in studies assessing its effects on health outcomes [file 1471-2458-10-415-S3.DOC]

**Additional file 3**

|  | **Study** | **Details of instrument** |
| --- | --- | --- |
|  | (Qiao, Taylor et al. 1989) | Self developed instrument, no validation reported |
|  | (Lubin, Qiao et al. 1990) | Self developed instrument, no validation reported |
|  | (Lubin, Li et al. 1992) | Self developed instrument, no validation reported |
|  | (Hsairi, Achour et al. 1993) | Self developed instrument, no validation reported |
|  | (Bedwani, ElKhwsky et al. 1997) | Self developed instrument, no validation reported |
|  | (Nuwayhid, Yamout et al. 1998) | Not reported |
|  | (Gupta, Boffetta et al. 2001) | Self developed instrument, no validation reported |
|  | (Habib, Mohamed et al. 2001) | Not reported |
|  | (Hazelton, Luebeck et al. 2001) | Not reported |
|  | (Medhat, Shehata et al. 2002) | Not reported |
|  | (Tamim, Musharrafieh et al. 2003) | Self developed instrument, no validation reported |
|  | (Al-Belasy 2004) | Not reported |
|  | (el-Sadawy, Ragab et al. 2004) | Not reported |
|  | (Natto, Baljoon et al. 2004) | Self developed instrument, no validation reported |
|  | (Baljoon 2005) | Self developed instrument, no validation reported |
|  | (Natto, Baljoon et al. 2005) | Self developed instrument, no validation reported |
|  | (Natto and Natto) | Self developed instrument, no validation reported |
|  | (Aghamolaei, Eftekhar et al. 2007) | Self developed instrument, no validation reported |
|  | (Ali and Ali 2007) | Not reported |
|  | (Nasrollahzadeh, Kamangar et al. 2008) | Self developed instrument, no validation reported for waterpipe tobacco use |
|  | (Tamim, Yunis et al. 2008) | Not reported |
|  | (Feng, Khyatti et al. 2009) | Self developed instrument, no validation reported |
|  | (Inhorn and Buss 1994) | Self developed instrument, no validation reported |

**References**

Aghamolaei, T., H. Eftekhar, et al. (2007). "Risk factors associated with Intrauterine Growth Retardation (IUGR) in Bandar Abbas." J. Med. Sci. **7**(4): 665-669.

Al-Belasy, F. A. (2004). "The relationship of "shisha" (water pipe) smoking to postextraction dry socket." Journal of Oral and Maxillofacial Surgery **62**(1): 10-14.

Ali, A. A. and A. A. Ali (2007). "Histopathologic changes in oral mucosa of Yemenis addicted to water-pipe and cigarette smoking in addition to takhzeen al-qat.[see comment]." Oral Surgery Oral Medicine Oral Pathology Oral Radiology & Endodontics **103**(3): e55-59.

Baljoon, M. (2005). "Tobacco smoking and vertical periodontal bone loss." Swedish Dental Journal - Supplement(174): 1-62.

Bedwani, R., F. ElKhwsky, et al. (1997). "Epidemiology of bladder cancer in Alexandria, Egypt: Tobacco smoking." International Journal of Cancer **73**(1): 64-67.

el-Sadawy, M., H. Ragab, et al. (2004). "Hepatitis C virus infection at Sharkia Governorate, Egypt: seroprevalence and associated risk factors." Journal of the Egyptian Society of Parasitology **34**(1 Suppl): 367-384.

Feng, B. J., M. Khyatti, et al. (2009). "Cannabis, tobacco and domestic fumes intake are associated with nasopharyngeal carcinoma in North Africa." Br J Cancer **101**(7): 1207-1212.

Gupta, D., P. Boffetta, et al. (2001). "Risk factors of lung cancer in Chandigarh, India." Indian Journal of Medical Research **113**: 142-150.

Habib, M., M. K. Mohamed, et al. (2001). "Hepatitis C virus infection in a community in the Nile Delta: Risk factors for seropositivity." Hepatology **33**(1): 248-253.

Hazelton, W. D., E. G. Luebeck, et al. (2001). "Analysis of a historical cohort of Chinese tin miners with arsenic, radon, cigarette smoke, and pipe smoke exposures using the biologically based two-stage clonal expansion model." Radiation Research **156**(1): 78-94.

Hsairi, M., N. Achour, et al. (1993). " Facteurs etiologiques du cancer bronchique primitif en Tunisie. ." La Tunisie Medicale **71**: 265-268.

Inhorn, M. C. and K. A. Buss (1994). "Ethnography, epidemiology and infertility in Egypt." Soc Sci Med **39**(5): 671-686.

Lubin, J. H., J. Y. Li, et al. (1992). "Risk of lung cancer among cigarette and pipe smokers in southern China." International Journal of Cancer **51**(3): 390-395.

Lubin, J. H., Y. L. Qiao, et al. (1990). "Quantitative evaluation of the radon and lung cancer association in a case control study of Chinese tin miners." Cancer Research **50**(1): 174-180.

Medhat, A., M. Shehata, et al. (2002). "Hepatitis C in a community in Upper Egypt: Risk factors for infection." American Journal of Tropical Medicine and Hygiene **66**(5): 633-638.

Nasrollahzadeh, D., F. Kamangar, et al. (2008). "Opium, tobacco, and alcohol use in relation to oesophageal squamous cell carcinoma in a high-risk area of Iran." Br J Cancer **98**(11): 1857-1863.

Natto, S., M. Baljoon, et al. (2004). "Tobacco smoking and gingival health in a Saudi Arabian population." Oral Health & Preventive Dentistry **2**(4): 351-357.

Natto, S., M. Baljoon, et al. (2005). "Tobacco smoking and periodontal bone height in a Saudi Arabian population." Journal of Clinical Periodontology **32**(9): 1000-1006.

Natto, S. B. and S. B. Natto "Tobacco smoking and periodontal health in a Saudi Arabian population." Swedish Dental Journal - Supplement(176): 8-52.

Nuwayhid, I. A., B. Yamout, et al. (1998). "Narghile (hubble-bubble) smoking, low birth weight, and other pregnancy outcomes." American Journal of Epidemiology **148**(4): 375-383.

Qiao, Y. L., P. R. Taylor, et al. (1989). "Relation of radon exposure and tobacco use to lung cancer among tin miners in Yunnan Province, China." American Journal of Industrial Medicine **16**(5): 511-521.

Tamim, H., U. Musharrafieh, et al. (2003). "Exposure of children to environmental tobacco smoke (ETS) and its association with respiratory ailments." Journal of Asthma **40**(5): 571-576.

Tamim, H., K. A. Yunis, et al. (2008). "Effect of narghile and cigarette smoking on newborn birthweight." BJOG: An International Journal of Obstetrics & Gynaecology **115**(1): 91-97 ANN: Please check whehter any related letters were published.
