# Supplementary material for: Survey instruments used in clinical and epidemiological research on waterpipe tobacco smoking: a systematic review
Source: BMC Public Health. 2010 Jul 13;10:415. doi: 10.1186/1471-2458-10-415 (PMC2912817; doi:10.1186/1471-2458-10-415)
Supplement: Additional file 4 — Validity of instruments in prevalence studies. Describes the validity of instruments to measure waterpipe tobacco smoking used in studies assessing its prevalence of use [file 1471-2458-10-415-S4.DOC]

**Additional file 4**

|  | **Study** | **Details of instrument** |
| --- | --- | --- |
|  | (Alam 1998) | Not reported |
|  | (Milaat, Al-Bar et al.) | Not reported |
|  | (Habib, Mohamed et al. 2001) | Self developed instrument, validation not reported |
|  | (Memon, Moody et al. 2000) | Not reported |
|  | (Baddoura and Wehbeh-Chidiac 2001) | Previously available instrument (Emile Roux questionnaire); validation not reported |
|  | (Medhat, Shehata et al. 2002) | Self developed instrument, validation not reported |
|  | (Al-Haddad, Hamadeh et al. 2003) | Self developed instrument, validation not reported |
|  | (Chaaya, Awwad et al. 2003) | Self developed instrument, validation not reported |
|  | (Gadalla, Aboul-Fotouh et al. 2003) | Self developed instrument, validation not reported |
|  | (Tamim, Terro et al. 2003) | Not reported |
|  | (Tamim, Musharrafieh et al. 2003) | Not reported |
|  | (Behbehani, Hamadeh et al. 2004) | Self developed instrument (based on a previously validated World Health Organization (WHO) questionnaire for health professionals(1984)), validation not reported |
|  | (Chaaya, El-Roueiheb et al. 2004) | Self developed instrument, validation not reported |
|  | (Chaaya, Jabbour et al. 2004) | Self developed instrument, validation reported |
|  | (el-Sadawy, Ragab et al. 2004) | Not reported |
|  | (Maziak, Eissenberg et al. 2004; Maziak, Fouad et al. 2004; Maziak, Hammal et al. 2004) | Self developed instrument (based on a standardized instrument from the WHO (IUTLD) (1998), and from previous questionnaires used in Syria (Maziak 2002)), validation not reported |
|  | (Zoughaib, Adib et al. 2004) | Self developed instrument, validation not reported |
|  | (Maziak, Ward et al. 2005) | Self developed instrument (based on a standardized instruments used in international settings as well as those used by Maziak et al in Syria (Maziak 2002; Maziak, Ward et al. 2005)), validation not reported |
|  | (Nisar, Billoo et al. 2005) | Self developed instrument, validation not reported |
|  | (Al-Turki 2006) | Not reported |
|  | (Rice, Weglicki et al. 2006) | Self developed instrument (based on previously reported validated instrument: Tobacco Use Questionnaire (TUQ)(Rice VH 2003)), validation not reported |
|  | (Ward, Eissenberg et al. 2006) | Not reported |
|  | (Ward, Vander Weg et al. 2006) | Not reported |
|  | (Mandil, Hussein et al. 2007) | Self developed instrument (based on previously developed validated instruments: WHO questionnaire(1983) and (GYTS) questionnaire(1998)), validation not reported |
|  | (Nisar, Qadri et al. 2007) | Self developed instrument, validation not reported |
|  | (Rice, Templin et al. 2007) | Self developed instrument (based on previously reported validated instrument: Tobacco Use Questionnaire (TUQ)(Rice VH 2003)), validation not reported |
|  | (Taha 2007) | Not reported |
|  | (Tamim, Al-Sahab et al. 2007) | Not reported |
|  | (Al-Mulla, Helmy et al. 2008) | Previously developed instrument (GYTS (2005)), no validation reported for waterpipe smoking |
|  | (Bachir and Chaaya 2008) | Not reported |
|  | (Jackson and Aveyard 2008) | Not reported |
|  | (Jawaid, Zafar et al. 2008) | Self developed instrument, validation not reported |
|  | (Parna, Usin et al. 2008) | Self developed instrument (1 question added to a previously validated instrument, the Health Behavior in School-aged Children survey (HBSC) of WHO(Maziak, Ward et al. 2005)), validation not reported |
|  | (Primack, Sidani et al. 2008) | Self developed instrument (8 questions added to the National College Health Assessment (NCHA) instrument(2004)), validation not reported |
|  | (Riachy, Rehayem et al. 2008) | Not reported |
|  | (Weglicki, Templin et al. 2007; Weglicki, Templin et al. 2008) | Self developed instrument (6 self questions added to Youth Risk Behavior Surveillance Survey (YRBSS)(Brener ND 2004)), validation not reported |
|  | (Primack, Walsh et al. 2009) | Self developed instrument (2 questions added to the Arizona Tobacco Survey (YTS) (July 2005)), validation not reported |
|  | (Carroll T 2008) | Not reported |

**References**

(1983). "Guidelines for the conduct of tobacco smoking surveys of the general population. Geneva, World Health Organization."

(1984). "World Health Organization (WHO). Guidelines for the Conduct of Tobacco-Smoking Surveys Among Health Professionals. World Health Organization. ." **WHO/SMO/84.1**.

(1998). World Health Organization. Guidelines for Controlling and Monitoring the Tobacco Epidemic. Geneva, Switzerland, WHO.

(2004). National College Health Assessment: ACHA-NCHA 2004, American College Health Association.

(2005). "The Global Tobacco Surveillance System Collaborating Group. The global tobacco surveillance system (GTSS): purpose, production and potential." J Sch Health **75**(1): 15-24.

(July 2005). Arizona Student Health Survey 2005 Methodology/Technical Report. Phoenix, AZ, Arizona Department of Education.

Al-Haddad, N., R. R. Hamadeh, et al. (2003). "Smoking among secondary-school boys in Bahrain: prevalence and risk factors." Eastern Mediterranean Health Journal **9**(1-2): 78-86.

Al-Mulla, A. M., S. A. Helmy, et al. (2008). "Prevalence of tobacco use among students aged 13-15 years in Health Ministers' Council/Gulf Cooperation Council Member States, 2001-2004." Journal of School Health **78**(6): 337-343.

Al-Turki, Y. A. (2006). "Smoking habits among medical students in Central Saudi Arabia." Saudi Medical Journal **27**(5): 700-703.

Alam, S. E. (1998). "Prevalence and pattern of smoking in Pakistan." JPMA - Journal of the Pakistan Medical Association **48**(3): 64-66.

Bachir, R. and M. Chaaya (2008). "Maternal smoking: Determinants and associated morbidity in two areas in Lebanon." Maternal and Child Health Journal **12**(3): 298-307.

Baddoura, R. and C. Wehbeh-Chidiac (2001). "Prevalence of tobacco use among the adult Lebanese population." Eastern Mediterranean Health Journal **7**(4-5): 819-828.

Behbehani, N. N., R. R. Hamadeh, et al. (2004). "Knowledge of and attitudes towards tobacco control among smoking and non-smoking physicians in 2 Gulf arab states." Saudi Medical Journal **25**(5): 585-591.

Brener ND, K. L., Kinchen S, Grunbaum J, Whalen L, Eaton D, et al. (2004). "Methodology of the Youth Risk Behavior Surveillance System." Morbidity & Mortality Weekly Report **53**: 1–13.

Carroll T, P. N., Perusco A. (2008). "Is concern about waterpipe tobacco smoking warranted ?" Australian and New Zealand Journal of Public Health. **32**(2): 181-U111.

Chaaya, M., J. Awwad, et al. (2003). "Demographic and psychosocial profile of smoking among pregnant women in Lebanon: public health implications." Maternal & Child Health Journal **7**(3): 179-186.

Chaaya, M., Z. El-Roueiheb, et al. (2004). "Argileh smoking among university students: a new tobacco epidemic." Nicotine & Tobacco Research **6**(3): 457-463.

Chaaya, M., S. Jabbour, et al. (2004). "Knowledge, attitudes, and practices of argileh (water pipe or hubble-bubble) and cigarette smoking among pregnant women in Lebanon." Addictive Behaviors **29**(9): 1821-1831.

el-Sadawy, M., H. Ragab, et al. (2004). "Hepatitis C virus infection at Sharkia Governorate, Egypt: seroprevalence and associated risk factors." Journal of the Egyptian Society of Parasitology **34**(1 Suppl): 367-384.

Gadalla, S., A. Aboul-Fotouh, et al. (2003). "Prevalence of smoking among rural secondary school students in Qualyobia governorate." Journal of the Egyptian Society of Parasitology **33**(3 Suppl): 1031-1050.

Habib, M., M. K. Mohamed, et al. (2001). "Hepatitis C virus infection in a community in the Nile Delta: risk factors for seropositivity." Hepatology **33**(1): 248-253.

Jackson, D. and P. Aveyard (2008). "Waterpipe smoking in students: prevalence, risk factors, symptoms of addiction, and smoke intake. Evidence from one British university." BMC Public Health **8**: 174.

Jawaid, A., A. M. Zafar, et al. (2008). "Knowledge, attitudes and practice of university students regarding waterpipe smoking in Pakistan." International Journal of Tuberculosis & Lung Disease **12**(9): 1077-1084.

Mandil, A., A. Hussein, et al. (2007). "Characteristics and risk factors of tobacco consumption among University of Sharjah students, 2005." Eastern Mediterranean Health Journal **13**(6): 1449-1458.

Maziak, W. (2002). "Smoking in Syria: profile of a developing Arab country." International Journal of Tuberculosis & Lung Disease **6**(3): 183-191.

Maziak, W., T. Eissenberg, et al. (2004). "Beliefs and attitudes related to narghile (waterpipe) smoking among university students in Syria." Annals of Epidemiology **14**(9): 646-654.

Maziak, W., F. M. Fouad, et al. (2004). "Prevalence and characteristics of narghile smoking among university students in Syria." International Journal of Tuberculosis & Lung Disease **8**(7): 882-889.

Maziak, W., F. Hammal, et al. (2004). "Characteristics of cigarette smoking and quitting among university students in Syria." Preventive Medicine **39**(2): 330-336.

Maziak, W., K. D. Ward, et al. (2005). "Mapping the health and environmental situation in informal zones in Aleppo, Syria: report from the Aleppo household survey." International Archives of Occupational and Environmental Health **78**(7): 547-558.

Maziak, W., K. D. Ward, et al. (2005). "Extent of exposure to environmental tobacco smoke (ETS) and its dose-response relation to respiratory health among adults." Respir Res **6**: 13.

Medhat, A., M. Shehata, et al. (2002). "Hepatitis c in a community in Upper Egypt: risk factors for infection." Am J Trop Med Hyg **66**(5): 633-638.

Memon, A., P. M. Moody, et al. (2000). "Epidemiology of smoking among Kuwaiti adults: prevalence, characteristics, and attitudes." Bulletin of the World Health Organization **78**(11): 1306-1315.

Milaat, W. A., H. S. Al-Bar, et al. "Preventive practices and non healthy behaviors among female university employees in Saudi Arabia." Bahrain Medical Bulletin 1999;21(3): 75-79.

Nisar, N., N. Billoo, et al. (2005). "Pattern of tobacco consumption among adult women of low socioeconomic community Karachi, Pakistan." JPMA - Journal of the Pakistan Medical Association **55**(3): 111-114.

Nisar, N., M. H. Qadri, et al. (2007). "A community based study about knowledge and practices regarding tobacco consumption and passive smoking in Gadap Town, Karachi." JPMA - Journal of the Pakistan Medical Association **57**(4): 186-188.

Parna, K., J. Usin, et al. (2008). "Cigarette and waterpipe smoking among adolescents in Estonia: HBSC survey results, 1994-2006." BMC Public Health **8**(1): 392.

Primack, B. A., J. Sidani, et al. (2008). "Prevalence of and associations with waterpipe tobacco smoking among US university students." Annals of Behavioral Medicine **36**(1): 81-86.

Primack, B. A., M. Walsh, et al. (2009). "Water-Pipe Tobacco Smoking Among Middle and High School Students in Arizona." Pediatrics **123**(2): e282-288.

Riachy, M., C. Rehayem, et al. (2008). "Are narghile smokers different from cigarette smokers?" Revue Des Maladies Respiratoires **25**(3): 313-318.

Rice, V. H., T. Templin, et al. (2007). "Collaborative research of tobacco use and its predictors in Arab and non-Arab American 9th graders." Ethnicity & Disease **17**(2): S19-S21.

Rice VH, T. T., Kulwicki A. (2003). "Arab American tobacco use: Four pilot studies." Preventive Medicine **37**: 492-498.

Rice, V. H., L. S. Weglicki, et al. (2006). "Predictors of Arab American adolescent tobacco use." Merrill-Palmer Quarterly-Journal of Developmental Psychology **52**(2): 327-342.

Taha, A. Z. A. (2007). "Prevalence of Risk-taking Behaviors." Bahrain Medical Bulletin **29**(4): 1-10.

Tamim, H., B. Al-Sahab, et al. (2007). "Cigarette and nargileh smoking practices among school students in Beirut, Lebanon." American Journal of Health Behavior **31**(1): 56-63.

Tamim, H., U. Musharrafieh, et al. (2003). "Exposure of children to environmental tobacco smoke (ETS) and its association with respiratory ailments." J Asthma **40**(5): 571-576.

Tamim, H., A. Terro, et al. (2003). "Tobacco use by university students, Lebanon, 2001." Addiction **98**(7): 933-939.

Ward, K. D., T. Eissenberg, et al. (2006). "The tobacco epidemic in Syria." Tobacco Control **15 Suppl 1**: i24-29.

Ward, K. D., M. W. Vander Weg, et al. (2006). "Waterpipe smoking among American military recruits." Preventive Medicine **43**(2): 92-97.

Weglicki, L. S., T. Templin, et al. (2007). "Tobacco use patterns among high school students: Do Arab American youth differ?" Ethnicity & Disease **17**((2 Suppl 3)): S3-22-S23-24.

Weglicki, L. S., T. N. Templin, et al. (2008). "Comparison of cigarette and water-pipe smoking by Arab and non-Arab-American youth." American Journal of Preventive Medicine **35**(4): 334-339.

Zoughaib, S. S., S. M. Adib, et al. (2004). "Prevalence and determinants of water pipe or narghile use among students in Beirut's southern suburbs." Journal Medical Libanais - Lebanese Medical Journal **52**(3): 142-148.
